# Supplementary material for: Concurrent selection of internal goals and external sensations during visual search
Source: Sci Adv. 2025 Nov 7;11(45):eadx8191. doi: 10.1126/sciadv.adx8191 (PMC12594192; doi:10.1126/sciadv.adx8191)
Supplement: Supplementary file 1 — Figs. S1 to S4 Supplementary Text [file sciadv.adx8191_sm.pdf]

Science Advances
AAAS

# Supplementary Materials for

## Concurrent selection of internal goals and external sensations during visual search

Baiwei Liu and Freek van Ede

Corresponding author: Baiwei Liu, b.liu@vu.nl; Freek van Ede, freek.van.ede@vu.nl

*Sci. Adv.* **11**, eadx8191 (2025)
DOI: 10.1126/sciadv.adx8191

**This PDF file includes:**

Figs. S1 to S4
Supplementary Text

SUPPLEMENTARY INFORMATION

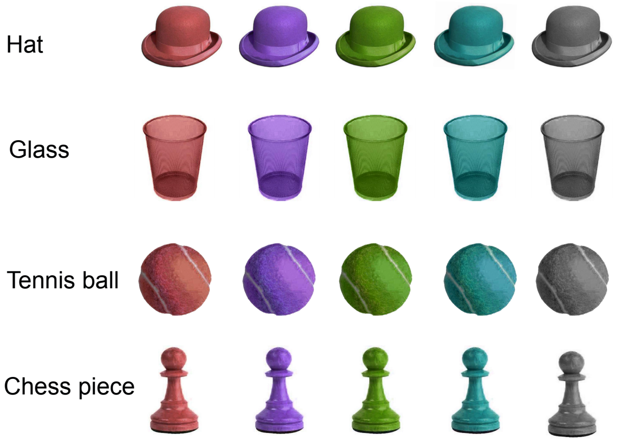


Figure S1. Stimuli used in Experiment 1.

## Control analyses

Here we rule out two possible low-level explanations for the observed concurrence of internal and external selection in our task. Because this involved follow-up control analyses on subsets of our trials, we focused these controls on Experiment 2 where we had more trials and participants available.

First, even though internal and external locations were independently manipulated in our task, it is conceivable that the reported concurrence is exclusively driven by those trials where the internal and external selection targets shared the same direction. To rule this out, we performed a follow-up analysis (control 1) where we exclusively considered trials in which the cued memory object and the matching search target were in different axes at perpendicular directions (e.g. memory object right, matching search target top). We still observed the same concurrent unfolding of our internal and external selection signals (**fig. S2A** jackknife analysis: $p = 0.81$, BF01 = 6.3).

Second, even though our tasks required selectively searching for the cued memory objects – not just any memory object – it is conceivable that our external-selection marker partly reflects attention being drawn to anything in the search display that looks familiar (i.e., to both memory objects). To rule out any influence of such familiarity, we performed another follow-up analysis (control 2). This time, we exclusively considered trials where the two memory objects *competed* along the same axis in the search display. This ensured that our external-selection signal – reflecting the difference in saccades toward vs. away from the *cued* search target – was exclusively sensitive to selection of the cued memory object (not just any memory object) on the screen. As with control 1, we still observed the same concurrent unfolding of our internal and external selection signals (**fig. S2B**; jackknife analysis: $p = 0.51$, BF01 = 5.3).

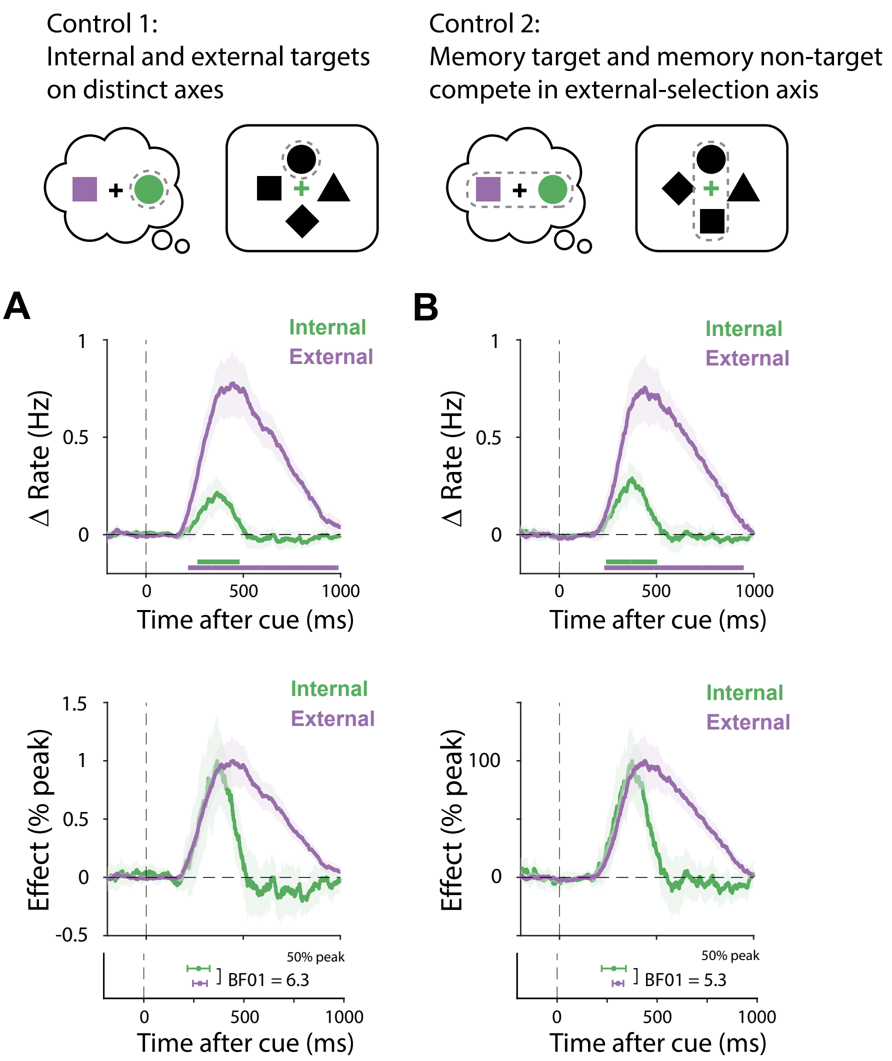


**Figure S2. Control analyses rule out low-level explanations for concurrent selection. A)** Time courses of spatial saccade modulations associated with internal and external selection in trials with perpendicular internal and external target directions. **B)** Time courses of spatial saccade modulations associated with internal and external selection in trials where the two memory objects competed along the same axis in the search display. Bottom panels show peak-normalised data together with onset-latencies calculated as the first sample reaching 50% of the peak. All time courses show mean values, with shading indicating ±1 SEM calculated across participants. The thick horizontal lines in the time course plots indicate significant temporal clusters (cluster-based permutation $P < 0.05$ (*71*)). Error bars on the onset latencies in the bottom panels were estimated using a Jackknife approach and show mean ± the 95% confidence interval. Bayes factors indicate evidence in favour of the null hypothesis of no difference.

## Sorting trials based on pre-cue EEG-alpha states

To examine the potential contribution of putative fluctuations in what memory object was spontaneously (by chance) in the focus of attention at the time of the cue, we performed an additional analysis on the data from experiment 2 where we had both eye-tracking and EEG data. In this analysis, we sorted the data based on pre-cue lateralization (in PO7/8) of posterior 8-12 Hz alpha-band activity – an established marker of the focus of attention in visual working memory (*42–46*).

For this, the epoched EEG time series (that we collected as part of Experiment 2) were transformed into a time–frequency representation using a short-time Fourier transform applied to Hanning-tapered data, as implemented in FieldTrip. Spectral power was estimated from 1 to 40 Hz in 1-Hz steps using a 300-ms sliding window, which advanced across the data in steps of 20 ms.

We included all trials in which the two memory objects were positioned on the left and right, and assessed 8-12 Hz alpha lateralization in PO7/8 within the 500-ms window before retrocue onset. Trials were categorized according to whether alpha power was lower contralateral to the subsequently cued object (consistent with neural activity states signalling that the to-be-cued memory object was more likely to be in the focus of attention) or lower contralateral to the non-cued object (consistent with neural activity states signalling that the other memory object was more likely to be in the focus of attention). We then analysed our primary gaze marker separately in these two types of trials. Outcomes are shown in **figure S3** below.

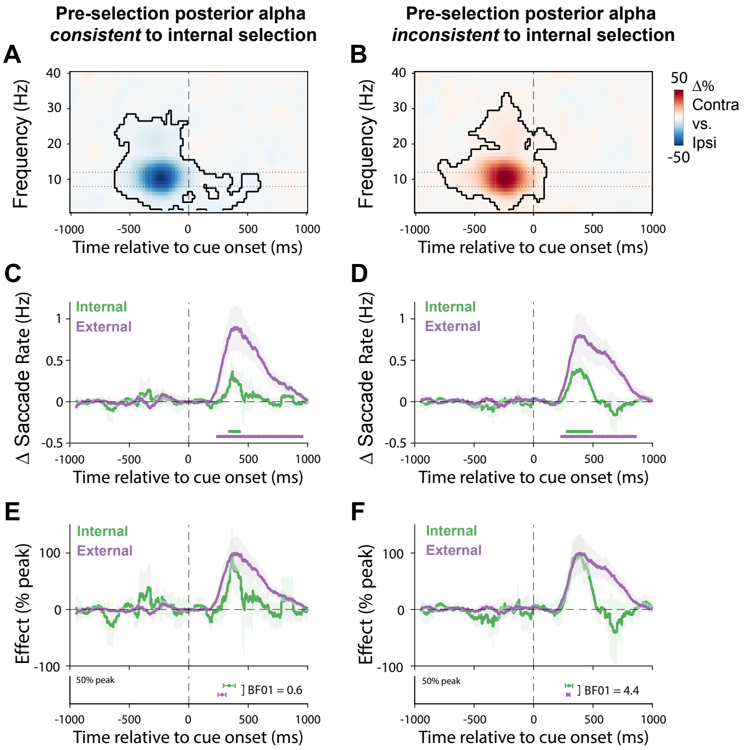


**Figure S3. Pre-cue alpha-based trial sorting reveals concurrent internal and external selection regardless of potential spontaneous prioritization.** We sorted the trials based on the pre-cue alpha power was lower contralateral to the subsequently cued memory object (**left column;** trials in which pre-cue alpha putatively signalled that the to-be-cued object was more likely to already be in focus at the time of the cue) or lower contralateral to the non-cued object (**right column;** trials in which pre-cue alpha putatively signalled that the other object was more likely to be in focus at the time of the cue). **(A, B)** Time-frequency results confirming the trials sorting. **(C, D)** Time courses of spatial saccade biases associated with internal and external selection. **(E, F)** Time course of the peak-normalised data together with onset-latencies calculated as the first sample reaching 50% of the peak. All time courses show mean values, with shading indicating ±1 SEM calculated across participants. The thick horizontal lines in the time course plots indicate significant temporal clusters (cluster-based permutation $P < 0.05$). Error bars on the onset latencies in the bottom panels were estimated using a Jackknife approach and show mean ± the 95% confidence interval.

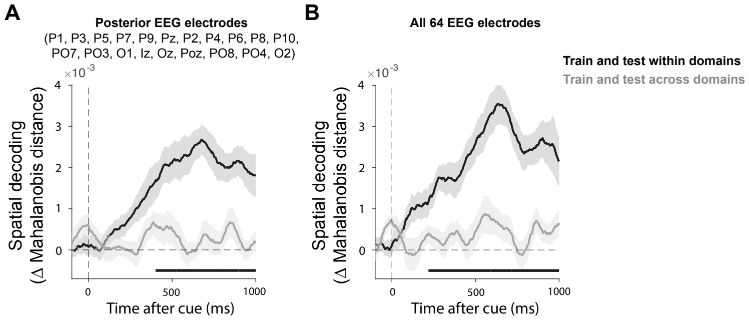


Figure S4. Complementary to Main Figure 3D, where the cross-domain decoding analysis was conducted in posterior electrodes (A), we also conducted the same analysis using all electrodes (B).
